# Draft genome assembly of the green-bronze dung beetle, *Onthophagus orpheus*

**DOI:** 10.1093/g3journal/jkag256

**Published:** 2026-09-16

**Authors:** Alexander J Bradshaw, Javier F Tabima, Erin L McCullough

**Affiliations:** Biology Department, Clark University, Worcester, MA 01610, United States; Biology Department, Clark University, Worcester, MA 01610, United States; Biology Department, Clark University, Worcester, MA 01610, United States

**Keywords:** *Onthophagus*, dung beetle, coleoptera, draft genome, genome assembly

## Abstract

Dung beetles (Coleoptera: Scarabaeinae) are ecologically important insects, yet genomic resources for this diverse lineage remain limited. Here, we present a high-quality genome assembly for *Onthophagus orpheus*, an understudied species that is abundant in urban forests in the eastern United States. The assembled genome is a scaffold-level assembly, with a high degree of genic completeness as assessed by Benchmarking Universal Single-Copy Ortholog analyses, indicating robust representation of conserved protein-coding genes. Structural and functional annotation recovered a comprehensive gene set consistent with expectations for coleopteran genomes. This genome assembly provides an important resource for future work on the behavioral ecology and population genetics of *Onthophagus orpheus*, specifically, and Scarabaeidae more broadly.

## Introduction

Dung beetles (Coleoptera: Scarabaeidae: Scarabaeinae) are a diverse and ecologically important group of insects that provide essential ecosystem functions, including dung degradation, pest and parasite control, nutrient recycling, soil aeration, and secondary seed dispersal (Nichols et al. 2008). The dung beetle genus *Onthophagus* is particularly diverse, with over 2,000 described species worldwide (Emlen et al. 2005b). Members of this genus exhibit complex mating behaviors and intense male–male competition that have favored the evolution of sexually selected horns (Halffter and Edmonds 1982; Emlen and Philips 2006). Because of this morphological and behavioral variation, *Onthophagus* dung beetles have emerged as a model for studying the genetic and developmental mechanisms underlying phenotypic plasticity and evolutionary lability (Emlen et al. 2005a; Moczek 2005).

Despite their ecological and evolutionary significance, genomic resources for onthophagine dung beetles remain limited. Chromosome-level genome assemblies are available for *Onthophagus taurus*, *O. sagittarius*, and *Digitonthophagus gazella* (Davidson and Moczek 2024), but genomic resources are still lacking for other *Onthophagus* species. Generating additional high-quality genome assemblies will support population genetics studies and conservation efforts for this ecologically important lineage.

Here, we present a high-quality draft genome assembly for the green-bronze dung beetle *Onthophagus orpheus (*Fig. 1A*)*. The species is native to the eastern United States and is the most abundant species in urban forests in central Massachusetts (McCullough et al. 2025). The assembly was generated using highly accurate PacBio HiFi sequencing. The resulting *O. orpheus* draft genome improves the genetic toolkit for this important genus and will facilitate comparative studies across Scarabaeinae.

## Results

### Sequencing coverage, genome assembly, and contiguity

Genome sequencing depth was sufficient to support a high-quality assembly, including 1,274,784 Pacbio Hifi reads representing 13.48 Gb (13,476,460,947 bp) of raw read output. Additionally, 99.45% of the assembly exhibited normal coverage, indicating that nearly the entire assembly fell within a ±2 standard deviation of the genome-wide median coverage. Assembly accuracy was further supported by k-mer-based evaluation, which estimated 79.2% k-mer completeness and a consensus quality value of 59.9, corresponding to a very low base-level error rate. Structural correctness metrics were also assessed using classification of regional assembly quality (CRAQ), which provides two measurements: R-AQI (read-based Assembly Quality Index) to quantify read support for accuracy and completeness, and S-AQI (structure-based Assembly Quality Index), which measures structural correctness by detecting potential misassemblies (Li et al. 2023). The genome assembly of *O. orpheus* achieved R-AQI = 93.9 and S-AQI = 97.7, respectively, indicating an agreement between the assembly accuracy and the underlying sequencing data (Table 1, Fig.1).

**Fig. 1.**
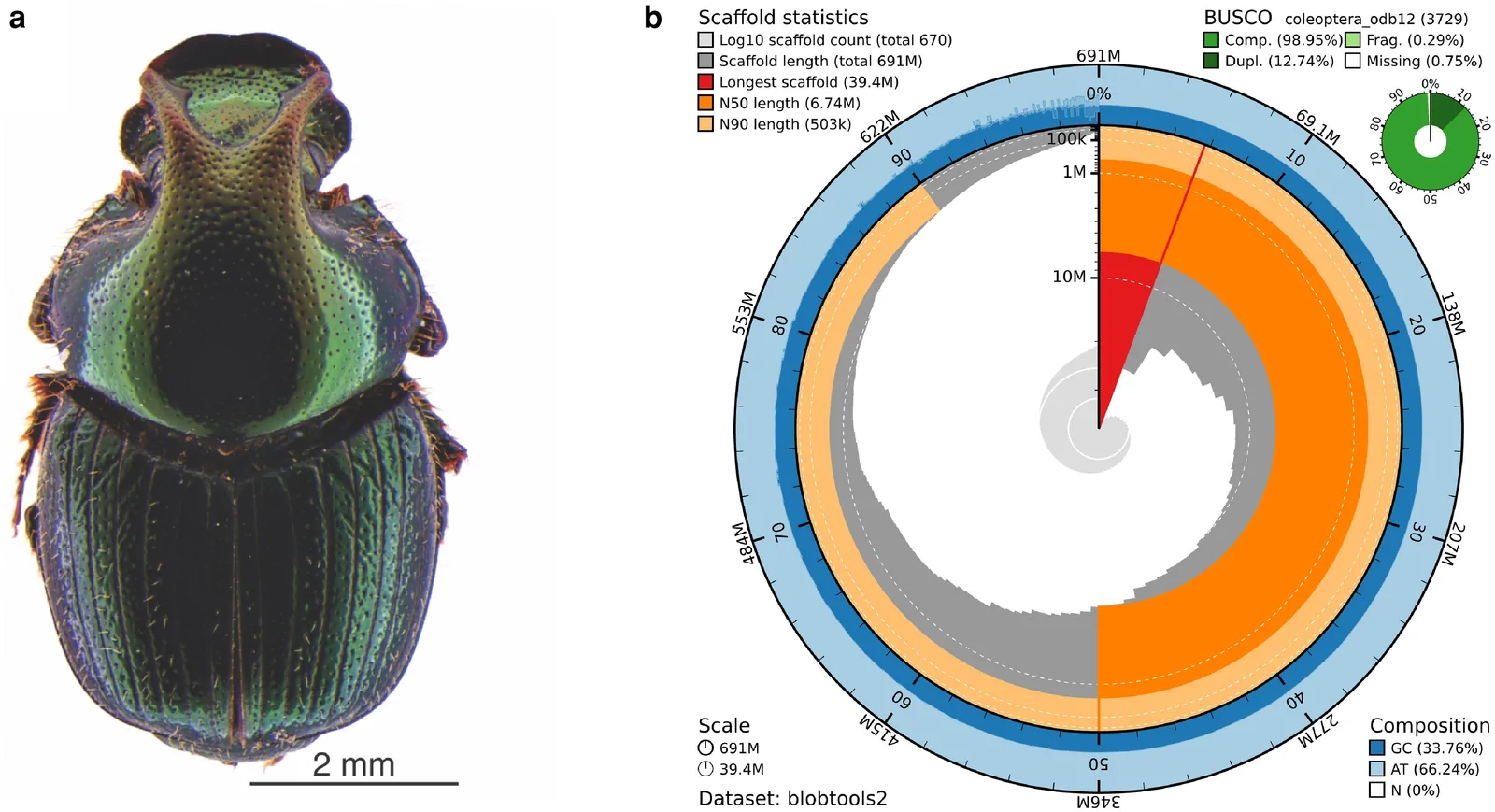
*Onthopagus orpheus* and genome summary. a) Dorsal view of a large *Onthophagus orpheus* male. Photo credit: Erin L. McCullough. b) Snail plot summarizing the genome assembly, including statistics on base composition size as well as BUSCO results derived from the coleoptera_odb12 database.

### Estimated completeness and genome size

The *O. orpheus* genome was assembled to a total length of 691.4 Mb, with the final assembly comprising 670 scaffolds, of which 667 exceed 10 kb, indicating a low degree of fragmentation. Assembly contiguity was high, with a scaffold N50 of 6.74 Mb and an N90 of 0.503 Mb, and the largest scaffold spanning 39.4 Mb. These metrics reflect strong long-range continuity and effective resolution of repetitive and structurally complex regions. Assembly completeness was also assessed using Benchmarking Universal Single-Copy Orthologs (BUSCO) with the coleoptera_odb12 dataset (*n* = 3,729). A total of 3,690 BUSCOs (98.95%) were recovered as complete, including 3,215 single-copy and 475 duplicated orthologs. Only 11 BUSCOs (0.3%) were fragmented, and 28 (0.8%) were missing (Fig. 1B). These results indicate that the assembly captures the vast majority of conserved beetle genes and is highly complete at the gene level. All total statistics are reported in Table 1. Genome size was estimated by generating a k-mer frequency histogram from raw sequencing reads and modeling the distribution with GenomeScope 2 (Ranallo-Benavidez et al. 2020). The fitted model closely matched the observed k-mer distribution, supporting the inferred genome characteristics. The estimated diploid genome size was approximately 420.3 Mb, with 23.5% unique sequence content and an estimated sequencing error rate of 0.416% (Fig. 2).

**Fig. 2.**
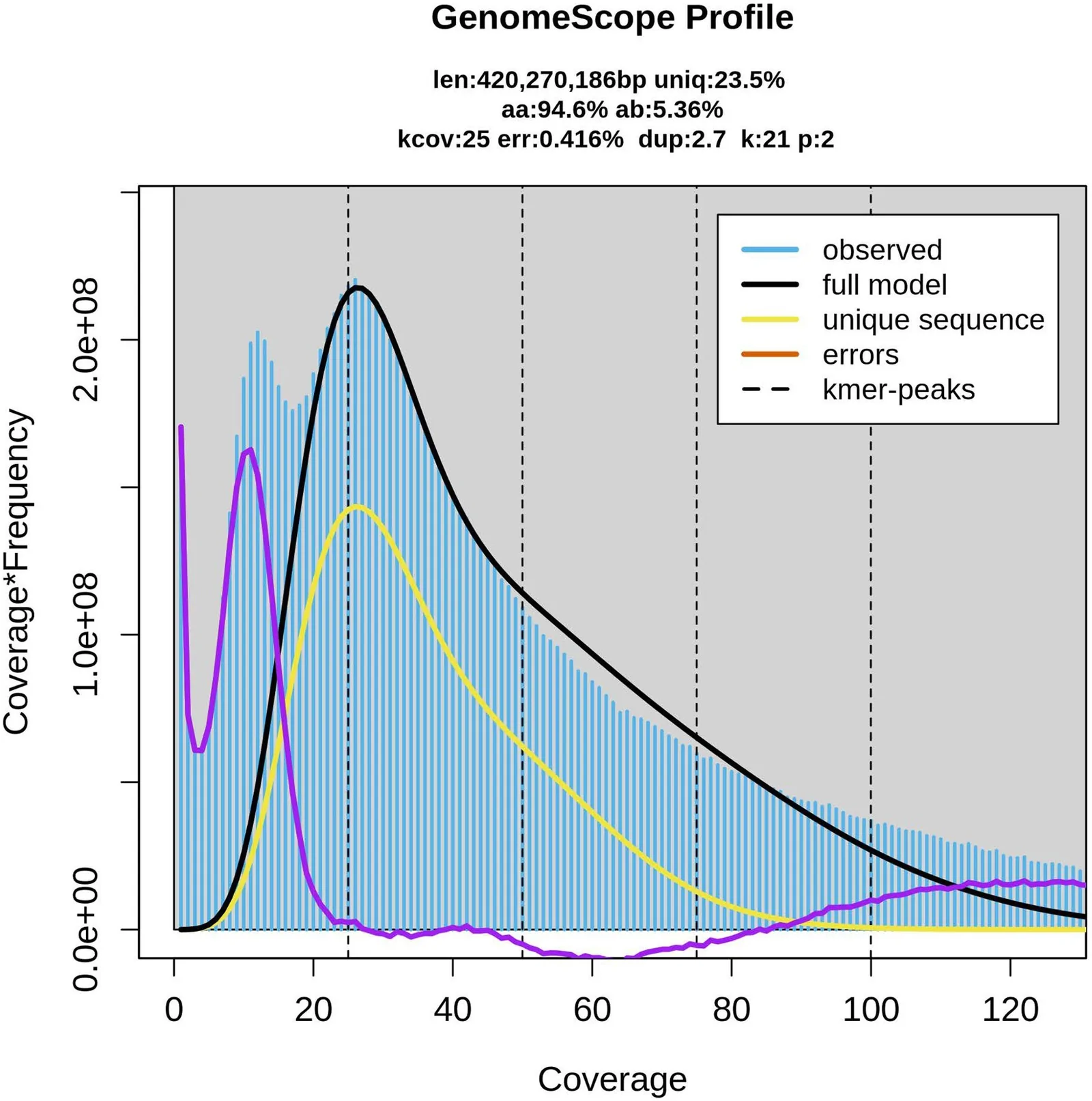
GenomeScope k-mer histogram profile used to estimate genome size. The observed k-mer frequency distribution generated from PacBio HiFi reads was modeled using GenomeScope 2 to estimate genome size (len), homozygous and heterozygous k-mer content (aa/ab), repeat content (dup), and sequencing error. The *x*-axis represents k-mer coverage depth, and the *y*-axis represents the number of distinct k-mers observed at each coverage level. The fitted GenomeScope 2 model is shown in black over the observed histogram (blue), with unique sequence content represented in yellow and model components associated with sequencing error shown in purple. Major peaks corresponding to heterozygous and homozygous k-mer classes are indicated by dotted lines.

**Table 1.** Genome assembly statistics and structural annotation for *Onthophagus orpheus.*

Metric	Value
Scaffolds	670
Scaffolds > 10 kb	667
Assembly size (MB)	691.4
Assembly N50 (MB)	6.74
Assembly N90 (MB)	0.503
Largest contig (bp)	39,430,979
Scaffolds >50 kb	518
% genome in scaffolds >50 kb	99.39%
GC content (%)	33.8%
Ambiguous bases (Ns)	0
BUSCO_db	coleoptera_odb12
BUSCO total genes	3729
BUSCO complete genes	3,690 (99%)
BUSCO complete single-copy genes	3,215 (86%)
BUSCO fragmented	11(0.2%)
BUSCO missing	28(0.7%)
merqury kmer completeness (%)	79.1993%
merqury qv (phred)	59.9008
CRAQ R-AQI	93.86228757
CRAQ S-AQI	97.65696959
Coverage normal (%)	99.4473%
Telomeric ends	0
Telomeric ends (%)	0%
Telomere to telomere scaffolds	0
Predicted genes	24,170
Predicted transcripts	23,317
Total exons	77,592
Total repeat families	4,022

### Gene content, telomeric analysis, and repetitive elements

Structural annotation of the *O. orpheus* genome identified 24,840 predicted protein-coding genes, represented by 23,317 transcripts and 80,476 exonic regions. Analysis of telomeric features detected no telomeric repeat motifs using the ancestral insect motif “TTAGG” at scaffold termini, and no telomere-to-telomere scaffolds were recovered. Despite the absence of detectable telomeric ends, assembly contiguity was high, with 518 scaffolds exceeding 50 kb, accounting for 99.39% of the assembled genome (Table 1, Fig. 3).

**Fig. 3.**
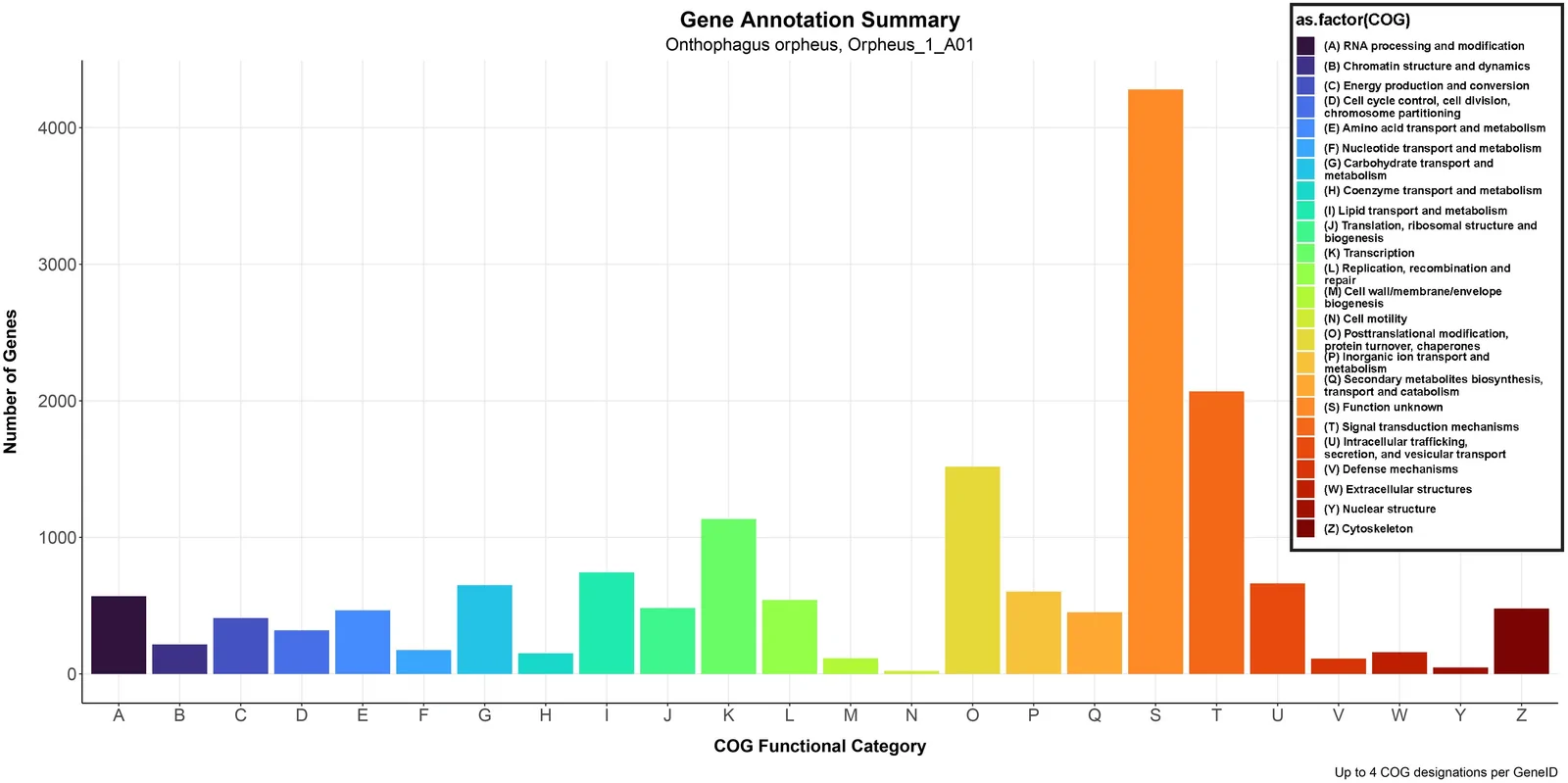
Distribution of predicted protein-coding genes across clusters of orthologous groups (COG) functional categories. Functional annotation of predicted protein-coding genes was performed using eggNOG-mapper, and genes were classified into COG functional categories. Bars represent the total number of genes assigned to each COG category shown along the *x*-axis.

Repetitive DNA analysis identified 4,022 repeat families across the genome. The assembled genome has a GC (guanine and cytosine) content of 33.8% and contains no ambiguous bases (Ns). Together, these results indicate a well-resolved gene set and repeat landscape suitable for downstream comparative and evolutionary analyses in Scarabaeinae.

### Functional annotation

Functional annotation of the *O. orpheus* genome was performed using a combination of similarity-based and domain-based approaches (see methods), resulting in putative functional assignments of predicted genes. In total, 24,174 protein-coding genes were predicted. Of these, 16,493 genes (68.2%) were assigned functional annotations based on eggNOG orthology, while 14,613 genes (60.4%) contained identifiable InterPro domains, and 12,603 genes (52.1%)) were associated with Pfam protein families. Gene ontology terms were assigned to 12,409 genes (51.3%), reflecting broad coverage of molecular functions, biological processes, and cellular components (Fig. 3). Additionally, transmembrane and secreted proteins were predicted, and a large number were associated with membrane localization and transport: 4,040 proteins (16.7%) were predicted to contain transmembrane domains, and 2,398 proteins (9.9%) were predicted to be secreted. This highlights that *O. orpheus* has numerous gene families potentially involved in extracellular signaling, digestion, and environmental interaction.

Enzymatic gene families relevant to insect physiology and metabolism were also well recovered. Prediction and annotation identified 925 putative proteases and 306 carbohydrate-active enzymes, including glycoside hydrolases and carbohydrate esterases, which likely contribute to nutrient processing and digestion. Collectively, these results indicate that the *O. orpheus* genome annotation captures a diverse and functionally rich gene repertoire. All annotation data have been provided in the Supplementary data.

## Discussion

This genome assembly provides a high-quality genomic resource for *Onthophagus orpheus*, an ecologically important dung beetle within the subfamily Scarabaeinae. The large, contiguous assembly and high BUSCO completeness indicate that the majority of protein-coding genes and conserved genomic regions are well represented, supporting the use of this genome as a reliable reference for downstream analyses. As such, the *O. orpheus* genome will be valuable for future comparative genomic studies within Scarabaeidae and Coleoptera, where sparse taxon sampling has constrained inference of genome evolution. Comparative analyses leveraging this genome can identify lineage-specific expansions, contractions, and structural variation associated with dung-feeding specialization, detoxification, and sensory adaptation, ecological processes known to drive diversification in beetles (McKenna et al. 2019).

The generation of a high-quality reference genome for *O. orpheus* also enables us to pursue future population-level analyses. High-quality reference genomes are essential for accurately mapping resequencing data, detecting selection, and reconstructing demographic history (Ellegren 2014), a growing research area within entomology (Raszick et al. 2021; Webster et al. 2023; Franzem et al. 2025). Dung beetles are sensitive to anthropogenic disturbance, including agricultural intensification, habitat fragmentation, and climate change, which can strongly influence population structure and adaptive variation (Davis et al. 2001; Nichols et al. 2007). This genome enables the identification of genomic regions associated with local adaptation, stress tolerance, and dispersal, and provides a framework for future conservation-genomic studies in dung beetles.

The *O. orpheus* genome assembly adds to a small but growing number of genomic resources for the speciose genus *Onthophagus*. This genus has emerged as a model system for studying sexual selection because of its remarkable diversity in horn morphology, condition-dependent trait expression, and alternative reproductive tactics. However, chromosome-level assemblies are currently available for only three species: *O. taurus*, *O. sagittarius*, and *Digitonthophagus* (formerly *Onthophagus*) *gazella* (Davidson and Moczek 2024). The availability of a reference genome for *O. orpheus* will facilitate comparative studies on the genomic architecture underlying horn development and horn polymorphisms (Kijimoto et al. 2013; Casasa et al. 2017), as well as investigations on mating systems and patterns of polyandry in natural populations (McCullough et al. 2017).

More broadly, this work improves the taxonomic representation of assembled beetle genomes and contributes to the study of insect genome evolution. Beetles exhibit extensive variation in genome size, repeat composition, and chromosomal organization, and comparative analyses suggest that transposable elements and gene duplication play major roles in shaping coleopteran genomes (Peters et al. 2017; McKenna et al. 2019). The estimated genome size predicted here for *O. orpheus* (∼420.3 Mb) exceeds that reported for *O. taurus* (∼291 Mb, GCA_036711975.1) but is substantially smaller than the ∼831 to 838 Mb genomes estimated for *Onthophagus binodis*. This variability has been thought to be due to the expansion of highly repetitive regions (Nadolski et al. 2026), of which we identified 4,022 families. It is possible that the evolution of repetitive genomic elements, or the ability to suppress repetitive elements, could be a route for future investigation across the genome, or considerable genome size variation within the genus *Onthophagus*. The *O. orpheus* assembly contributes to these efforts by providing a scarabaeine reference that can be integrated into large-scale comparative datasets to understand how ecological specialization and developmental novelty influence genome architecture.

Together, this genome assembly improves genomic representation within Scarabaeidae and provides a resource for future studies in understanding the biology of the genus *Onthophagus.* As additional dung beetle genomic data become available, the *Onthophagus orpheus* genome will serve as a key comparative reference for investigating the genomic basis of ecological specialization, developmental innovation, and the evolution of sexually selected traits in an ecologically impactful beetle lineage.

## Methods

### Specimen acquisition, genomic DNA extraction, and sequencing

Beetles were collected with pitfall traps on 2024 June 20 from Hadwen Arboretum (42.258°N, −71.832°W). We followed the basic salt-extraction method of Aljanabi and Martinez (1997) to extract genomic DNA from adult male *Onthophagus orpheus*. The legs, head, and thorax of each individual were homogenized in 350 µL sterile salt homogenizing buffer (50 mM Tris-HCl pH 8.0, 100 mM EDTA, 100 mM NaCl, and 1% SDS) and 2.5 µL of 20 mg/mL proteinase K using a pellet pestle. The samples were incubated at 65 °C with intermittent vortexing for 15 min, cooled on ice, and mixed with 150 µL 5 M NaCl. Samples were vortexed vigorously for 10 s and spun down at 13,000 rpm for 10 min. The supernatant was transferred to fresh tubes, mixed with 500 µL of isopropanol, and centrifuged at 13,000 rpm for 10 min. The pellet was washed with 70% ethanol, dried, and finally resuspended in 50 µL TE buffers. Genomic DNA (13.56 µg) from one individual was sent to the UMass Chan Medical School Deep Sequencing Core for PacBio sequencing.

### Data quality control, genome assembly, quality assessment, and estimation of genome size

Genome assembly of *Onthophagus orpheus* was performed using the PacBio HiFi Assembly pipeline developed by the Harvard Informatics Group (https://github.com/harvardinformatics/pacbio_hifi_assembly), which incorporates a series of optimized tools for processing and assembling PacBio HiFi reads using the highly accurate Hifiasm (Cheng et al. 2021) genome assembler into a reproducible Snakemake workflow (Köster and Rahmann 2012). The pipeline automates read correction, consensus generation, and assembly graph construction, providing a reproducible framework for high-quality long-read genome assemblies. All analyses were executed using a singularity container cloned directly from the public repository and run with default parameters appropriate for diploid eukaryotic genomes to ensure software consistency and reproducibility. The resulting primary contig (Orpheus_1_A01.p_ctg.fa) served as the base assembly for subsequent quality control and contamination removal. Genome size estimation was performed by creating a kmer-based histogram of the raw reads using Jellyfish v.2.2.10 (Marçais and Kingsford 2011) with a kmer size of 21. This kmer histogram table was then used in conjunction with GenomeScope2 v.2.0.1 (Ranallo-Benavidez et al. 2020), using a kmer value of 21 and a ploidy of 2, reflecting the diploid nature of the sequenced specimen.

### Quality assessment, contamination removal, preprocessing, and repetitive-region masking

Initial assembly quality and structural integrity were assessed using QUAST v5.2.0 (Gurevich et al. 2013), comparing the *O. orpheus* assembly to the *Onthophagus taurus* (GCF_036711975.1) and *Onthophagus sagittarius* (GCA_036711965.1) reference genomes, as well as the raw unpolished PacBio HiFi assembly (Supplementary data). Visualization of the whole genome and BUSCO scores was performed using the blobtk plot -v snail function of the blobtookit (Challis et al. 2020) suite of tools. Metrics, including contig count, N50, and total assembly length, were recorded to evaluate polishing and contamination-removal performance and are provided in the Supplementary data.

Following assembly quality analysis, the genome was screened for potential adapter contamination and contamination from foreign organisms using the NCBI Foreign Contamination Screen (FCS-GX) toolset v0.5.5 (Astashyn et al. 2024). The goal was to identify and remove nontarget sequences before downstream annotation. Screening was performed against the National Center for Biotechnology Information (NCBI) *Genome Taxonomy Database*, using the Onthophagus taxonomic identifier (NCBI: txid166331) as the reference to remove contamination.

The contamination-free assembly was then preprocessed for annotation using the “clean” function in Funannotate v1.8.17 (Palmer and Stajich 2020) to remove potential low-quality and redundant scaffolds, as well as those shorter than 500 bp. Repetitive elements were identified using RepeatModeler v2.0.7 (Flynn et al. 2020), which performs de novo repeat discovery by integrating the RepeatScout (Price et al. 2005) and RECON (Bao and Eddy 2002) algorithms. This analysis identified 4,022 repeat families, which were subsequently used as a species-specific repeat library (Supplementary data) to mask repetitive regions in the assembly prior to gene prediction and annotation. This sample-specific repeat database was then combined with the Dfram (Storer et al. 2021) repeat element database release 3.9 and used for repeat masking before gene prediction.

### Gene prediction and functional annotation

For integrated gene prediction, the “predict” function of Funannotate v1.8.17 was employed using the masked repetitive-region assembly. Here, we used a combination of Augustus (Stanke et al. 2006), GenMark-ES (Borodovsky and Lomsadze 2011), GlimmerHMM (Majoros et al. 2004), and semi-HMM-based nucleic acid parser (SNAP) ( (Korf 2004) gene prediction software with the flags –busco_seed_species tribolium and –protein_evidence from the protein sequences of the *O. taurus* reference (GCF_036711975.1) to guide gene prediction. High-quality predictions were then determined using EVidenceModeler (Haas et al. 2008), with equal weight given to all gene prediction methods and repeat regions incorporated into the EVidenceModeler (EVM)step (−repeats2evm) to improve gene model placement in masked regions. After prediction, the resulting Augustus model parameters were archived with the Funannotate “species” function to preserve the optimized *O. orpheus* species profile for future gene prediction experiments, which are also provided in the Supplementary data.

Functional annotation of predicted gene models was performed using the Funannotate “annotate function,” with the insecta_odb9 BUSCO database guiding quality evaluation. Functional annotation was then performed with InterProScan5 5.59_91.0 (Jones et al. 2014) and eggNOG-mapper v2.1.12 (Cantalapiedra et al. 2021). Finally, secreted and transmembrane protein prediction was performed with Phobius v1.01 (Käll et al. 2004) and the fast algorithm of SignalP 6 (Teufel et al. 2022).

### Quantification and visualization of the final assembly and annotations

Quality assessment using the PAQman v1.2.0 (O’Donnell et al. 2025) pipeline leverages multiple commonly used, well-established bioinformatics tools to comprehensively assess genome assemblies. This includes: contiguity is assessed using QUAST (Gurevich et al. 2013); BUSCO scores (Simão et al. 2015); completeness and base-level error rates estimation by Merqury (Rhie et al. 2020); structural correctness metrics using CRAQ (Li et al. 2023); coverage metrics generated through read mapping and utilities using Minimap2 (Li 2018) for long and short read alignment, Samtools for alignment processing (Danecek et al. 2021), and Bedtools (Quinlan and Hall 2010) for genomic coverage calculations. Finally, telomerality features are derived from sequence processing with Seqtk v.1.5 (https://github.com/lh3/seqtk) and Bedtools (Quinlan and Hall 2010). Visualization of gene ontology results from the function annotation was performed by mutating the data frame output from Funannotate using the R package tidyverse v2.0.0 (Wickham et al. 2019), and then plotting with ggplot2 v4.0.0 (Wickham 2016). Final assembly statistics are presented in Table 1, and all PAQman outputs are provided in Supplementary data.

## Data Availability

All raw sequencing reads have been deposited in the NCBI Sequencing Read Archive (SRA) under Bioproject number PRJNA1395262. Raw sequencing reads are deposited on the SRA under the accession number SRR36630064. The draft genome assembly and annotations are deposited in NCBI under biosample SAMN54354319 and accession number JCDJZF000000000. Supplementary data, including full annotation results, Augustus gene prediction mode, full assembly statistics, GenomeScope analysis, and repetitive elements library, can be accessed from the Open Science Framework repository at https://doi.org/10.17605/OSF.IO/CE2AB. Supplemental material available at G3 online.
